# Supplementary material for: Flexible temperature-pressure dual sensor based on 3D spiral thermoelectric Bi2Te3 films
Source: Nat Commun. 2024 Mar 21;15:2521. doi: 10.1038/s41467-024-46836-1 (PMC10958038; doi:10.1038/s41467-024-46836-1)
Supplement: Supplementary file 1 — Supplementary Information [file 41467_2024_46836_MOESM1_ESM.pdf]

Supplementary Information

Flexible temperature-pressure dual sensor based on 3D spiral thermoelectric Bi_2Te_3 films

Hailong Yu[1,2], Zhenqing Hu[1,2], Juan He[1,2], Yijun Ran[1,2], Yang Zhao[2], Zhi Yu[1,2,*], Kaiping Tai[1,2,3*]

[1]School of Materials Science and Engineering, University of Science and Technology of China, Shenyang, 110016, China

[2]Shenyang National Laboratory for Materials Science, Institute of Metal Research, Chinese Academy of Sciences, Shenyang, 110016, China

[3]Liaoning professional technology innovation center for integrated circuit thermal management, Shenyang 110016, China

[*]Correspondence and requests for materials should be addressed to K. P. T. (email: kptai@imr.ac.cn), Z. Y. (email: zyu@imr.ac.cn)

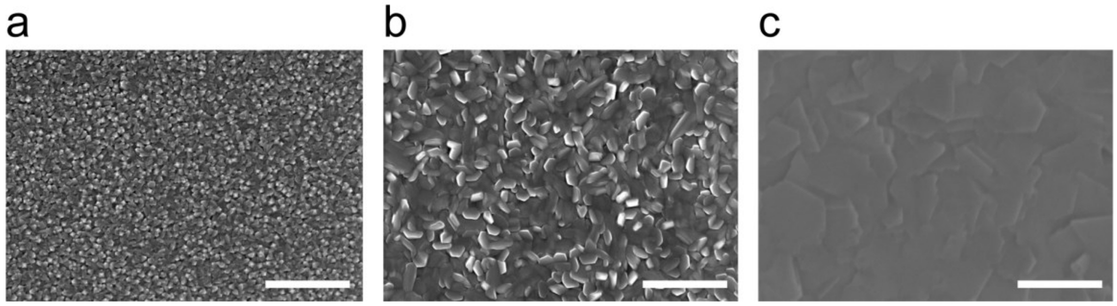

Supplementary Figure 1 | Surface SEM image of the Bi_2Te_3 film deposited at different temperatures. (a) 298 K (b) 473 K(c) 613 K. Scale bars, 1 μm (a, b, c)

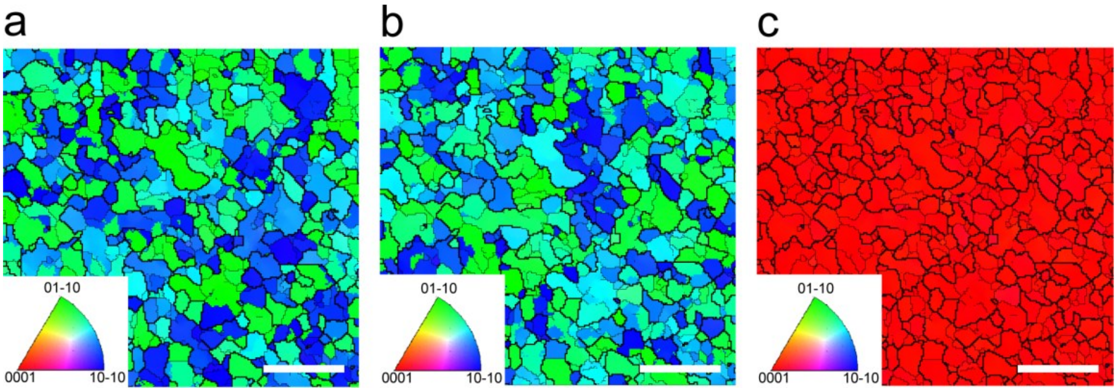

Supplementary Figure 2 | The EBSD image of the Bi_2Te_3 film deposited at 613 K.

(a) X_0 direction IPF image. (b) Y_0 direction IPF image. (b) Z_0 direction IPF image. Scale bars, 1 μm (a, b, c)

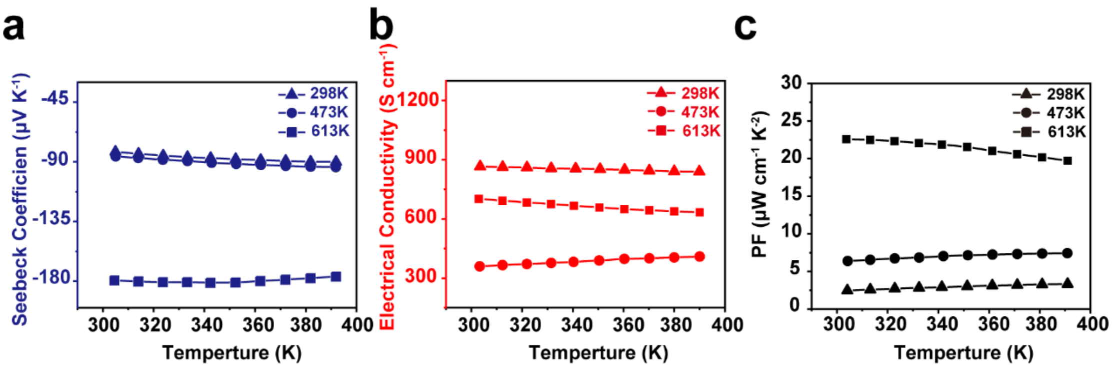

Supplementary Figure 3 | The thermoelectric performance of the film deposited at different temperatures. The temperature-dependent Seebeck Coefficient (a), Electrical Conductivity (b), and Power Factor (c) of those films. Source data are provided as a Source Data file.

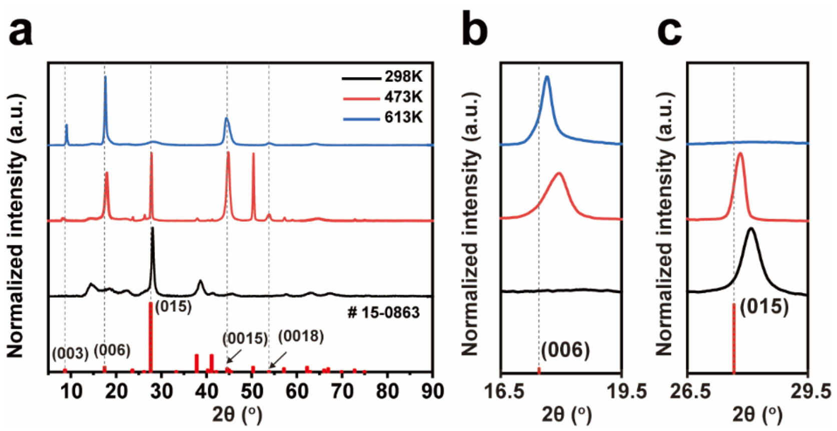

Supplementary Figure 4 | XRD spectrum of the Bi_2Te_3/PI (polyimide) films. (a) The films deposited at 298 K, 473 K, and 613 K. (b) Enlarge of the spectrum near (006) peak. (c) Enlarge of the spectrum near (015) peak. Source data are provided as a Source Data file.

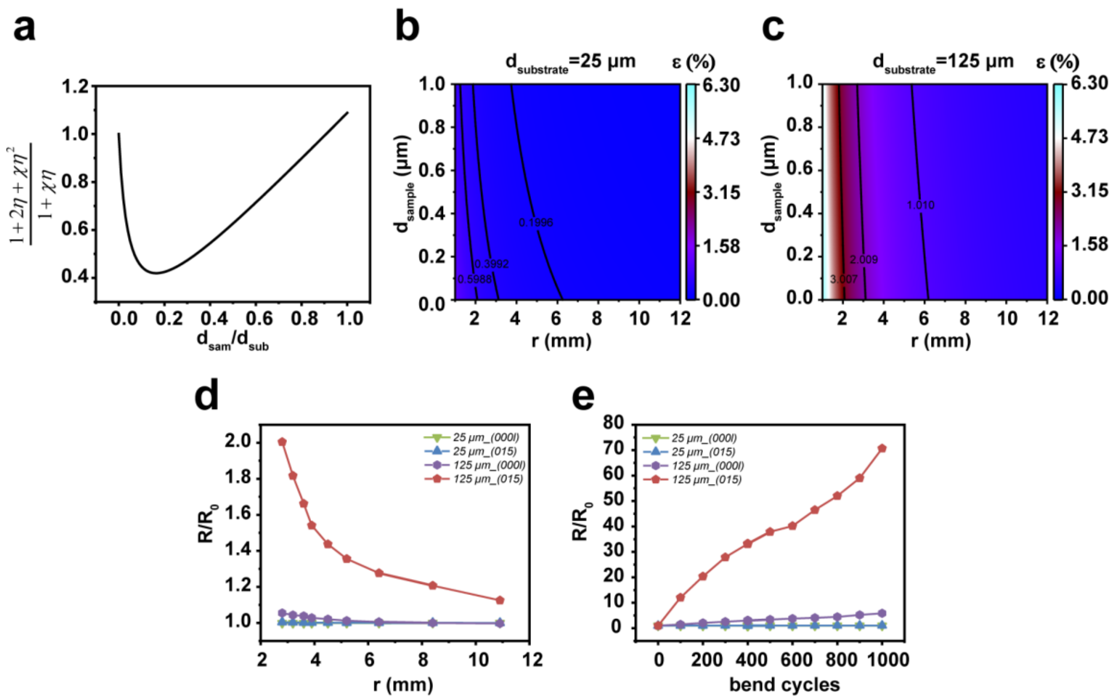

Supplementary Figure 5 | Estimation of strain during film bending and the flexibility test result of various Bi_2Te_3/polyimide films with the same *d_{sample}*. (a) The coefficient $\frac{1+2\eta+\chi\eta^2}{1+\chi\eta}$ as a function of $d_{sample}/d_{subtrate}$. (b-c) Stress nephogram at different film thicknesses and bending radii under 25 μm (b) and 125 μm (c) thick substrate, respectively. The solid black lines represent the isostrain lines. (d) Relative electrical resistance as a function of bending radius for the film. (e) The resistance change of film under 1000 cycles bend test at a bend radius of 2.8 mm. Legend Description: The lower triangle and superior triangle represent the sample with *(000l)*-texture and *(015)*-texture film deposited on a 25 μm thick substrate. The hexagons and pentagons represent the sample with *(000l)*-texture and *(015)*-texture film deposited on a 125 μm thick substrate. Source data are provided as a Source Data file.

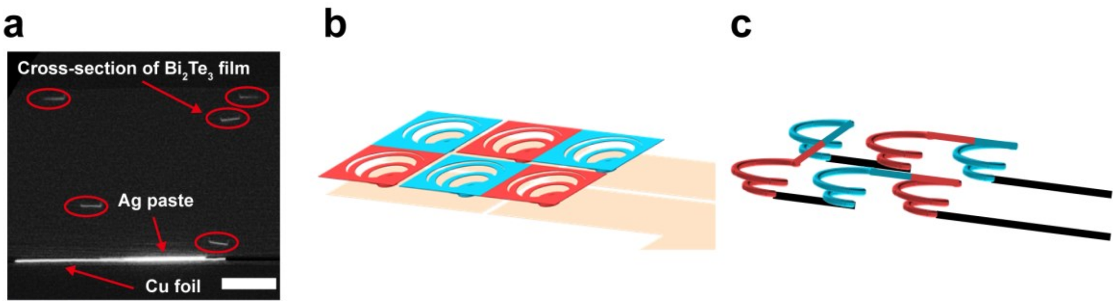

Supplementary Figure 6 | The spiral structure of the sensor and its electrical connection. (a) The cross-section of the spiral structure imaging by XRT. The red ellipse represents Bi_2Te_3 film, the bright line is Ag paste and the Cu foil. Scale bars, 250 μm. (b) Simplified schematic diagram of device electrical connections. (c) The interconnection details of the 3-pair device. The red spiral and blue spiral represent the Au film and the Bi_2Te_3 film, and the black line denotes the Cu foil used as an electrode.

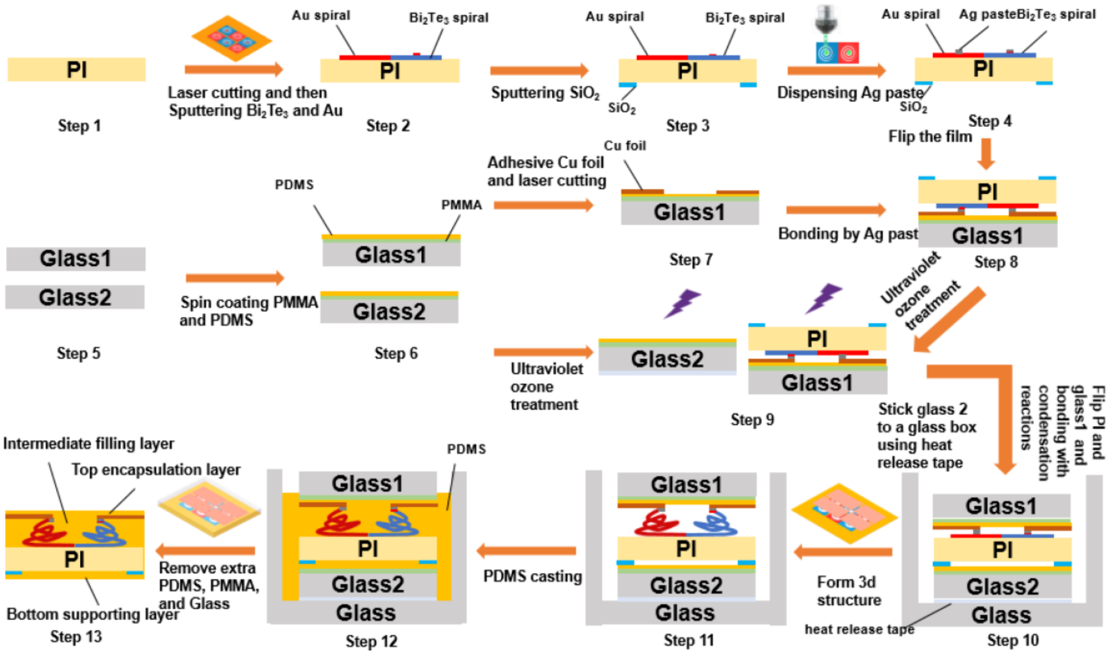

Supplementary Figure 7 | The manufacture steps of the pressure-temperature sensor.

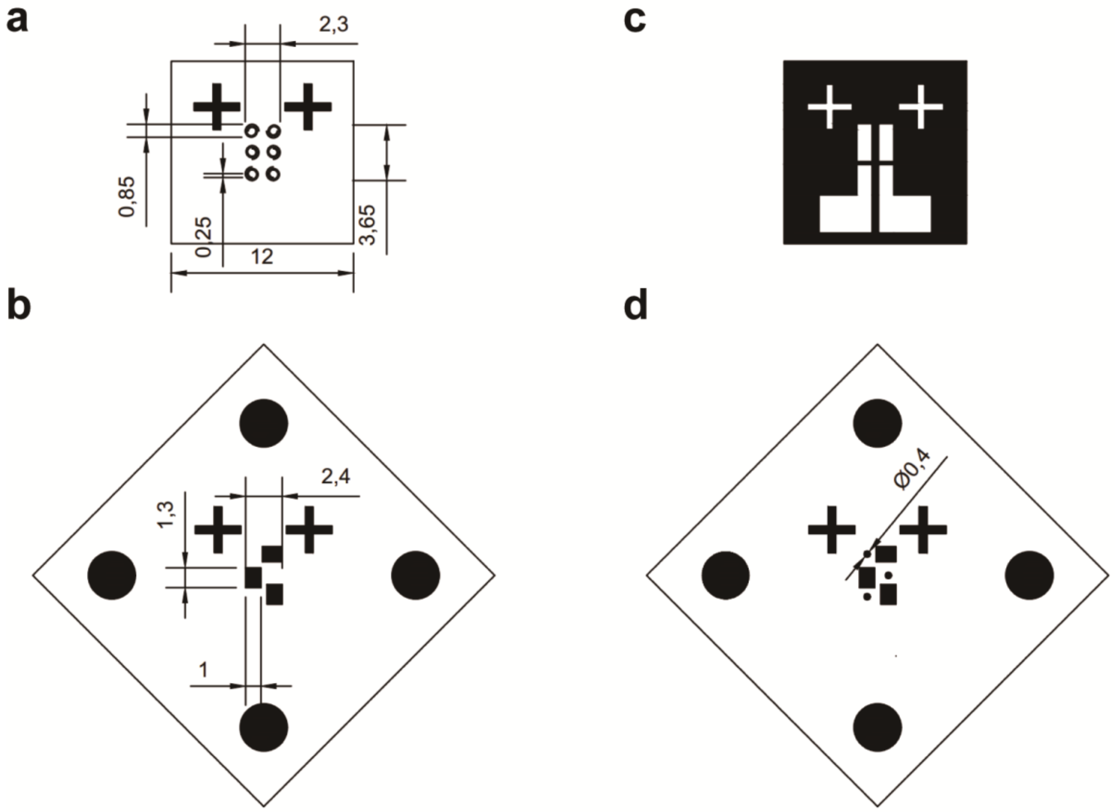

Supplementary Figure 8 | Additional pictures for a single sensor. The black area is what the laser has removed, and the round across and holes are designed for alignment. (a) The PI 2D spiral structure. (b) The metal mask for Bi_2Te_3. (c) The Cu foil electrode pattern. (d) The metal mask for Au film deposition.

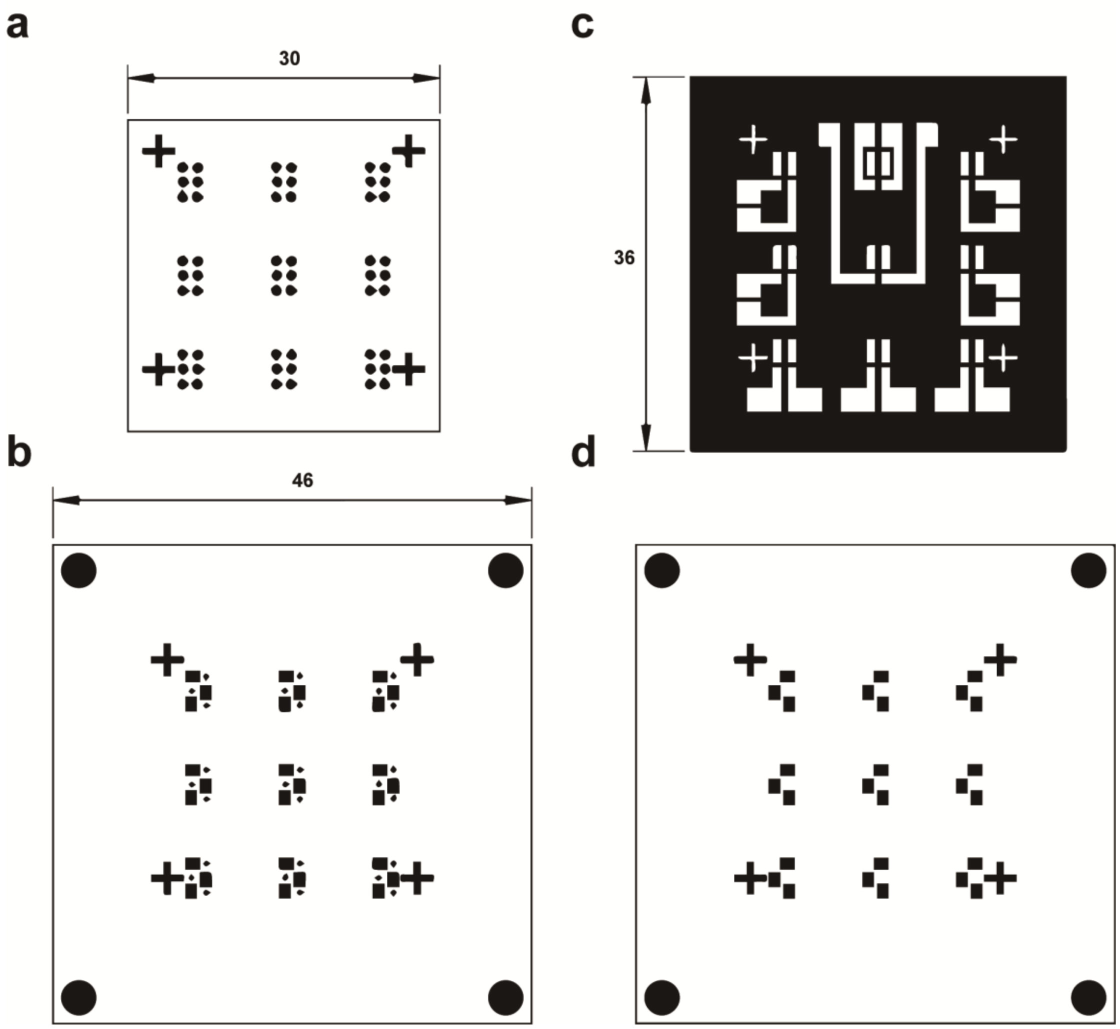

Supplementary Figure 9 | Additional pictures for the 3×3 sensor array. The black area is what the laser has removed, and the round across and holes are designed for alignment. (a) The PI 2D spiral structure. (b) The metal mask for Au film deposition. (c) The Cu foil electrode pattern. (d) The metal mask for Bi_2Te_3.

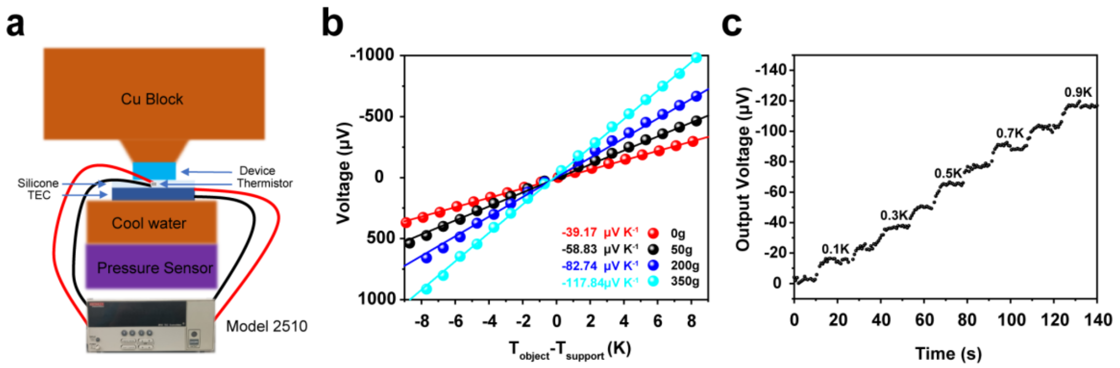

Supplementary Figure 10 | The contact thermal resistance affects the measurement of sensitivity. (a) The schematic diagram of the sensitivity test. (b) The output voltage as a function of the T_{object}-$T_{support}$ at different press situations. (c) The real-time response of the sensor to a temperature gradient of 0.1 K at a press of 350 g. Source data are provided as a Source Data file.

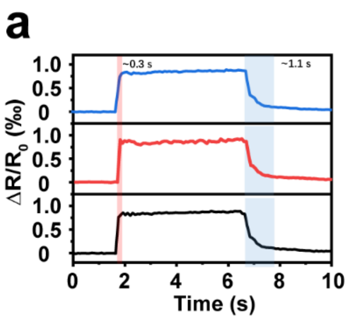

Supplementary Figure 11 | Pressure sensing response time of the sensor. The time-resolved response of the sensor to pressure stimuli with the red and blue zones corresponding to the response time and recovery time. The 3 types of color of the curve represent 3 times the test for the sensor. Source data are provided as a Source Data file.

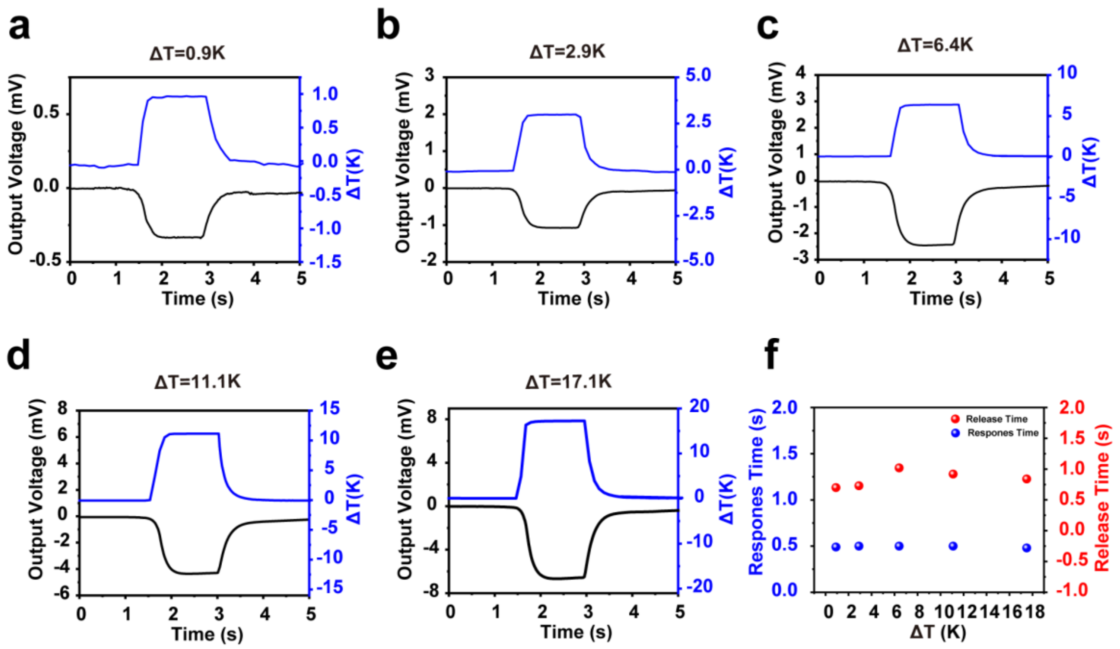

Supplementary Figure 12 | Temperature sensing response time of the sensor. (a-e) Voltage response to an input signal of different ΔT from 0.9 K to 17.1 K. The Blue curve represents the temperature signal, and the black curve represents the voltage response of the sensor. (f) Response time as a function of ΔT. Source data are provided as a Source Data file.

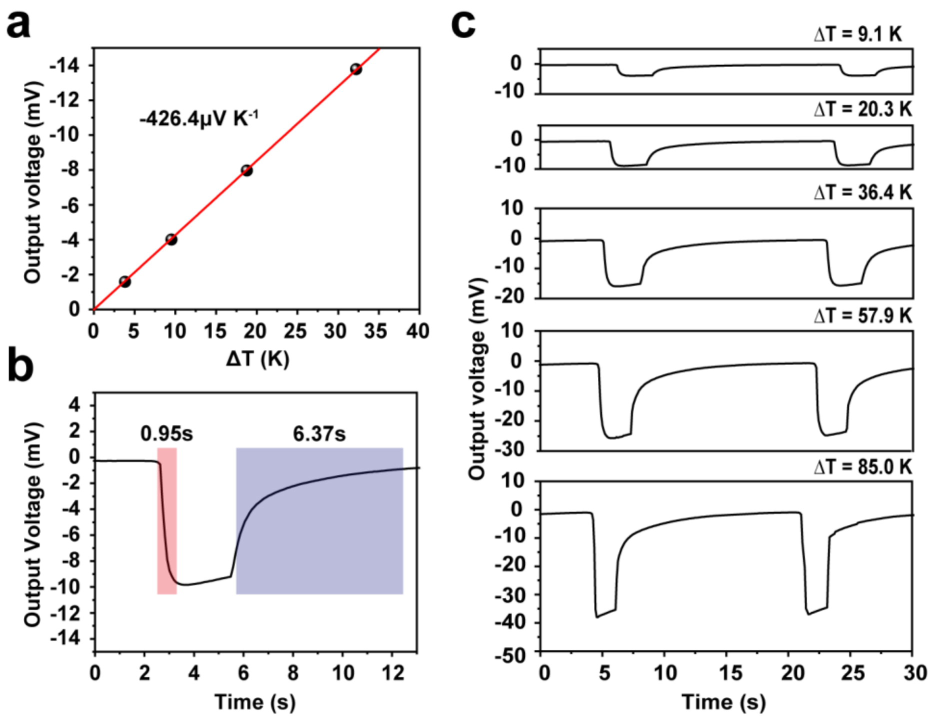

Supplementary Figure 13 | The sensing performance of the 1 mm thick pressure-temperature sensor. (a) The output voltage is a function of the sensor's temperature gradient. The red line is the fitted curve, showing a sensitivity of 426.4 μV K^{-1}. (b) The time-resolved response of the sensor to temperature stimuli with the red and purple zones corresponds to the response and relaxation time, respectively. (c) Voltage response to temperature difference stimuli from 9.1 K to 85.0 K. Source data are provided as a Source Data file.

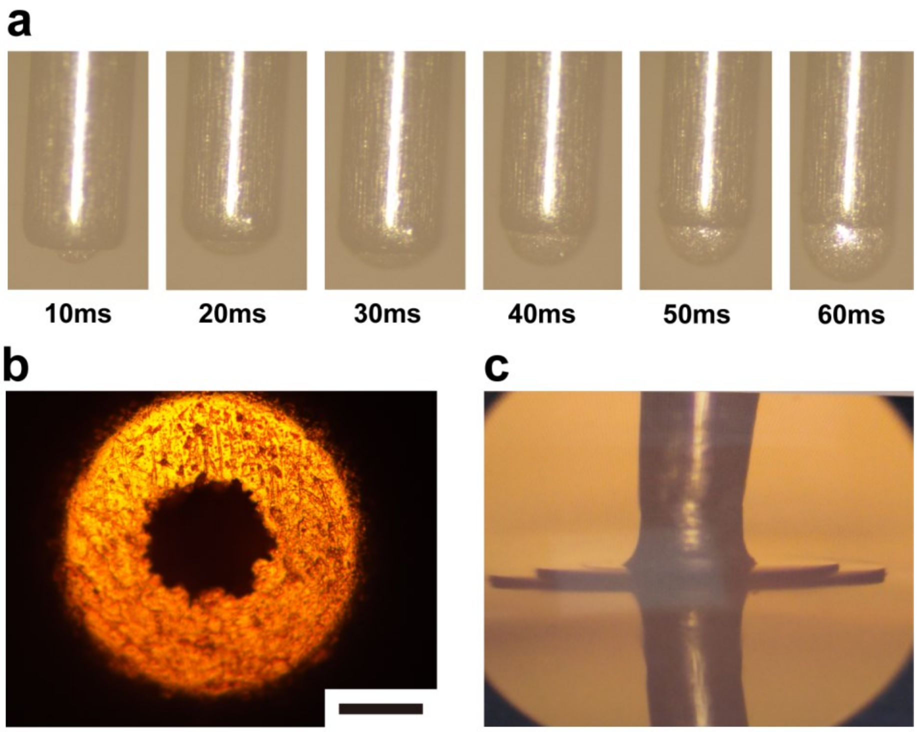

Supplementary Figure 14 | The image of the dispensing device. (a) Cross-sectional image of silver paste at 234 kPa pressure for different dispensing times. (b) Micrograph of the cross-section of a dispensing needle. Scale bar: 50 μm. (c) Cross-sectional image during dispensing.

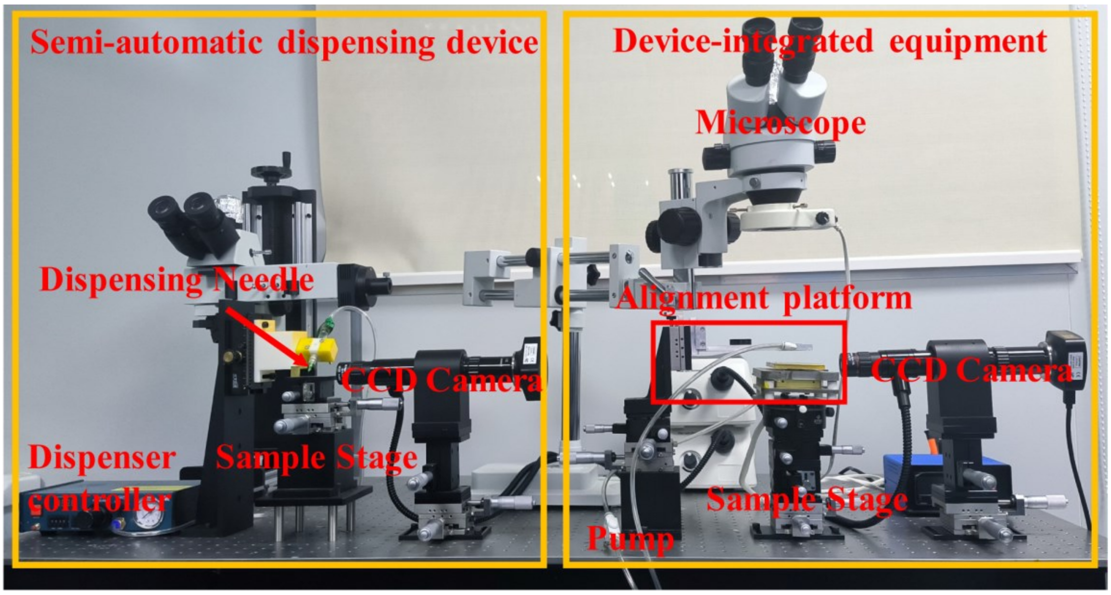

Supplementary Figure 15 | The lab-built semi-automatic dispensing device (left) and device-integrated equipment (right).

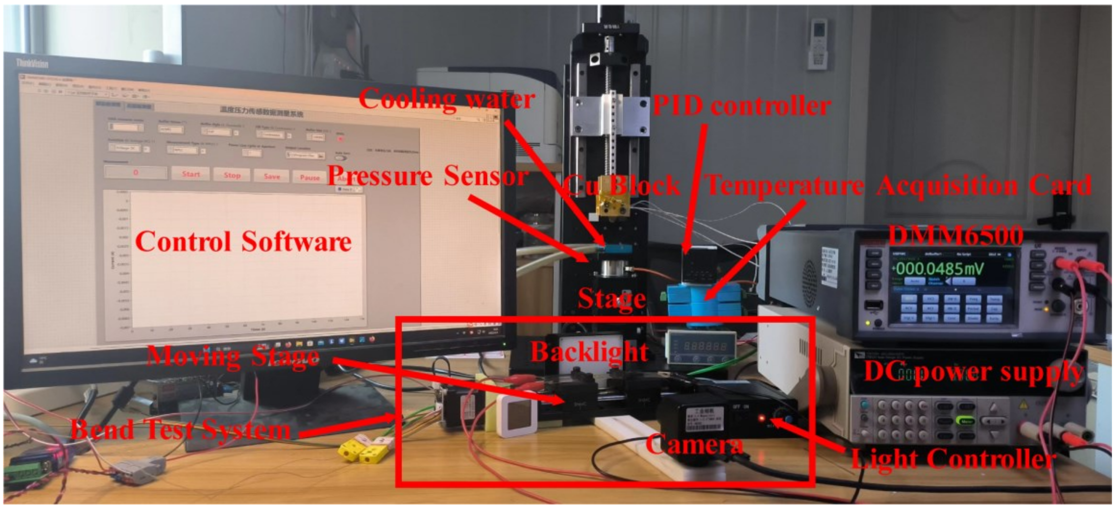

Supplementary Figure 16 | The lab-built temperature-pressure and flexible test system.

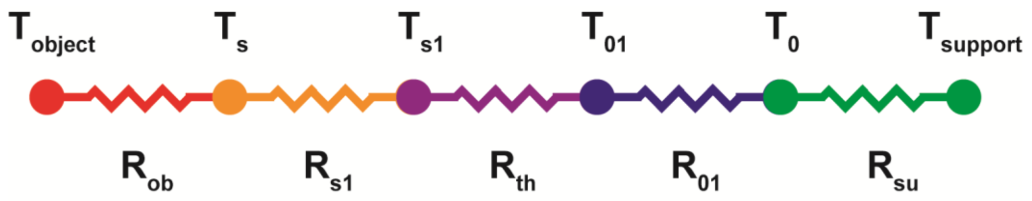

Supplementary Figure 17 | The one-dimensional heat conduction model.

Supplementary Table 1 | The carrier concentration and mobility of the Bi_2Te_3 film deposited in different temperature

Deposition temperature (K)	Carrier concentration (cm^{-3})	Mobility (cm^2 v^{-1} s^{-1})
**298**	**-5.1×10^{20}**	**2.4**
**473**	**-1.38×10^{20}**	**53.9**
**613**	**-4.54×10^{19}**	**117**

Supplementary Notes

Supplementary Note1 Material performance optimization

The *(000l)* textured Bi_2Te_3 demonstrated a superior thermoelectric performance due to the excellent electrical performance along the a-b plane. A flexible Bi_2Te_3 film was deposited on 25 μm thick polyimide using magnetron sputtering at 298 K, 473 K, and 613 K, and its SEM image is shown in Supplementary Figure 1. The grain size increases with the deposition temperature, and the surface of the film becomes smoother. At 613 K, the film exhibits an extraordinarily smooth surface without visible nano-pores. The presence of a large number of hexagonal and triangular grains indicates their stacking along the c-axis, which was confirmed by the XRD spectrum shown in Supplementary Figure 4a. Moreover, the XRD shows that the *(000l)* peak intensity is enhanced with the deposition temperature, indicating the intensity of *(000l)* texture becomes stronger. The Electron Back-Scattered Diffraction (EBSD) results also confirmed the presence of a very strong *(000l)* texture in the film deposited at 613 K, as shown in Supplementary Figure 2.

The thermoelectric performance also improved with increasing deposition temperature. As can be seen in the Supplementary table. 1, the carrier concentration decreases while the mobility increases with the temperature increase. This may be due to the reduced defect density at higher deposition temperatures, resulting in weaker electron scattering. Therefore, the higher mobility and lower concentration lead to the highest electric conductivity observed in the film deposited at 473 K, rather than at 613K, as shown in Supplementary Figure 3c. The temperature-dependent Seebeck coefficient is shown in Supplementary Figure 3a. The Seebeck coefficient is about -90 $\mu V\ K^{-1}$ for the film deposited at 298 K and 473 K and about -180 $\mu V\ K^{-1}$ for the film deposited at 613 K, which mainly depended on the carrier concentration. In addition, the power factor can be calculated, as shown in Supplementary Figure 3b, where the film deposited at 613 K exhibits the highest value of 22.6 $\mu W\ cm^{-2}\ K^{-1}$.

Supplementary Note 2 Strain analysis of the flexible test for Bi_2Te_3/polyimide films

One of the primary strategies for enhancing the flexibility of thin films is to minimize internal strain during film deformation. Therefore, the analysis of internal strain during film bending is of significant importance. When a deposited film and a compliant substrate have the same Young's modulus, the strain in the top bending sample (ε_b) is as follows,

$$\varepsilon_b = \frac{d_{sample} + d_{substrate}}{2r_b} \tag{9}$$

Where d_{sample} and $d_{substrate}$ are the thickness of film and polyimide, respectively, and r_b is the bending radius. In this scenario, a thicker film under the same bending radius will result in greater strain. But in our case, the modulus of Bi_2Te_3 and polyimide differs. The ε_b should be given by[1–3]:

$$\varepsilon_b = \frac{d_{sample} + d_{substrate}}{2r_b} \frac{1 + 2\eta + \chi\eta^2}{(1+\eta)(1+\chi\eta)} = \frac{d_{substrate}}{2r_b} \frac{1 + 2\eta + \chi\eta^2}{1+\chi\eta} \tag{10}$$

Where $\eta = d_{sample}/d_{substrate}$ and $\chi = Y_{sample}/Y_{substrate}$, $Y_{sample} = 54.2\ Gpa$ and $Y_{substrate} = 2.5\ Gpa$ denote Young's modulus of the film and substrate[4,5].

Supplementary Note 3 Device manufacture process

Supplementary Figure 7 shows the sensor's fabricating process, it contains several steps below:

Preparation of Bi_2Te_3/PI film for sensor manufacture:

1. A 25 μm PI film was cut into six 2D spiral structures using a femtosecond laser by loading a CAD image as shown in Supplementary Fig. 5a to the cutting program.

2. Depositing Bi_2Te_3 film using the metal mask as shown in Supplementary Figure5b, then the metal mask shown in Supplementary Fig. 5d was used to deposit Au film.

3. Sputtering SiO_2 film to the back side of the film completed in step 2.

4. Dispensing Ag pastes to the Au film located at the middle of every spiral for the electrical connection and physic bond of the next step.

Preparation of the auxiliary part for sensor manufacture:

5. Preparing 2 glasses named glass 1 and glass 2.

6. Spin coating PMMA and PDMS on glass 1 and glass 2.

7. Adhesiving 4.5 μm Cu foil to glass1 and cut according to Supplementary Figure. 5c to form the upper electrode of the sensor.

Assembly process of the sensor:

8. Align the silver in step 4 with the Cu foil in step 7 using the lab-built device-integrated equipment as shown in Supplementary Fig. 9, then press the PI and glass1 and heat at 140 K for 30 min to form strong electrical and physical connections between the spiral and Cu foil.

9. Put the part completed in step 8 and the glass 2 in step 6 into the UV-ozone cleaner to clean for 5 minutes for the next chemically bond step between the SiO_2 and the PDMS.

10. Sticking the glass 2 prepared in step 9 to an open glass box using heat release tape. Then the SiO_2 of the PI film clean in step 9 was bonded to the PDMS of the glass2 using the device-integrated equipment.

11. Glass 1 was absorbed by vacuum adsorption in the device-integrated equipment, and moved down the glass 2 to form the 3D spiral structure.

12. Casting PDMS and curing for 12 hours.

13. Cutting the extra PDMS, heating to release the sensor, and then clean with acetone to remove the PMMA.

The sensor array was fabricated using the same methods above, except for the sensor size, electrode shape, and metal mask used differently as shown in Supplementary Fig. 6.

Supplementary Note 4 Thermal resistance analysis

We build a simple one-dimensional heat conduction model (Supplementary Figure. 17) based on the Fourier heat equation to analyze the heat conduction of the sensor as below:

$$R_{tot} = R_{ob} + R_{s1} + R_{th} + R_{01} + R_{su} \tag{1}$$

$$\frac{T_{object} - T_s}{R_{ob}} = \frac{T_s - T_{s1}}{R_{s1}} = \frac{T_{s1} - T_{01}}{R_{th}} = \frac{T_{01} - T_0}{R_{01}} = \frac{T_0 - T_{support}}{R_{su}} \tag{2}$$

$$T_0 = \frac{R_{su}}{R_{tot}}\left(T_{ob} - T_{su}\right) + T_{su} \tag{3}$$

$$T_s = \frac{R_{01} + R_{s1} + R_{th} + R_{su}}{R_{tot}}\left(T_{ob} - T_{su}\right) + T_{su} \tag{4}$$

$$T_{01} = \frac{1}{R_{tot}}\left[\left(R_{01} + R_{su}\right)T_{ob} + \left(R_{ob} + R_{s1} + R_{th}\right)T_{su}\right] \tag{5}$$

$$T_{s1} = \frac{1}{R_{tot}}\left[\left(R_{01} + R_{su} + R_{th}\right)T_{ob} + \left(R_{ob} + R_{s1}\right)T_{su}\right] \tag{6}$$

$$S = \frac{\alpha\left(T_{s1} - T_{01}\right)}{T_s - T_0} = \frac{R_{th}}{R_{01} + R_{s1} + R_{th}}\alpha = \left(1 - \frac{R_{01} + R_{s1}}{R_{01} + R_{s1} + R_{th}}\right)\alpha = A\alpha \tag{7}$$

$$S_{ob\_su} = \frac{\alpha\left(T_{s1} - T_{01}\right)}{T_{object} - T_{support}} = \frac{R_{th}}{R_{tot}}\alpha = \frac{R_{01} + R_{s1} + R_{th}}{R_{tot}}S \tag{8}$$

Where the T_{object} is the temperature of the object to be detected, T_s is the temperature of the upside surface of the sensor, T_{s1} is the temperature of the upside surface of the active materials, T_{01} is the temperature of the downside surface of the active materials, T_0 is

the temperature of the downside surface of the sensor, and $T_{support}$ is the temperature of the object which the sensor attaches on. The R_{ob} and R_{su} are the contact thermal resistance in the upside surface of the sensor and the downside surface of the sensor, respectively, R_{s1} and R_{01} are the parasitic thermal resistance of the sensor, and R_{th} is the effective thermal resistance of the sensor. S is the actual sensitivity of the sensor, α is the Seebeck coefficient of active materials, A is the effective temperature coefficient, and S_{ob_su} is the measurement sensitivity.

In this model, we ignored the convection and radiation because the sensor is assumed in contact with the object. According to the heat flow balance, we can get equation (2), and then by combining equation (1), we can derive equation (3-8) to express the temperature of every interface of the sensor during the work. So, even though the actual sensitivity only depends on the sensor itself, the inevitable external thermal resistance (R_{s1} and R_{su}) will affect the measurement accuracy of the sensitivity as seen in equations (7) and (8).

Supplementary Fig. 10b shows how the contact thermal resistance affects the measurement sensitivity. Under the pressure of 0 g, the measurement sensitivity (S_{ob_su}) is only -39.17 μV/K, which is not the intrinsic parameter of the sensor. With the increase in pressure, the measurement sensitivity, S_{ob_su}, increases. When the pressure reaches 350g, the S_{ob_su} reaches -117.84 μV K^{-1}. It is worth noting that we employed T_{ob} and T_{su} (temperature of the copper contacted with the sensor's upper surface and temperature of the thermally conductive silicone contacted with the sensor's lower surface) rather than T_s and T_0 (temperatures of the upper and lower surfaces of the sensor) to calculate the ΔT. Due to the presence of unavoidable R_{ob} and R_{su}, as mentioned in equation (8), the measurement sensitivity, $S_{ob\text{-}su}$, is less than the actual sensitivity, S.

Supplementary References

1. Jin, Q. *et al.* Flexible Carbon Nanotube-Epitaxially Grown Nanocrystals for Micro-Thermoelectric Modules. *Adv. Mater.* 2304751 (2023) doi:10.1002/adma.202304751.
2. Jin, Q. *et al.* Flexible layer-structured Bi2Te3 thermoelectric on a carbon nanotube scaffold. *Nat. Mater.* **18**, 62–68 (2019).
3. Suo, Z., Ma, E. Y., Gleskova, H. & Wagner, S. Mechanics of rollable and foldable film-on-foil electronics. *Appl. Phys. Lett.* **74**, 1177–1179 (1999).
4. Huang, B.-L. & Kaviany, M. *Ab initio* and molecular dynamics predictions for electron and phonon transport in bismuth telluride. *Phys. Rev. B* **77**, 125209 (2008).
5. Huang, J. *et al.* Polyimide/POSS nanocomposites: interfacial interaction, thermal properties, and mechanical properties. *Polymer* **44**, 4491–4499 (2003).
